# Nuclear respiratory factor 1 regulates super enhancer-controlled SPIDR to protect hepatocellular carcinoma cells from oxidative stress

**DOI:** 10.1186/s12876-024-03183-1

**Published:** 2024-03-04

**Authors:** Baowang Liu, Jian Dou, Jinglin Cao

**Affiliations:** Department of Hepatobiliary Surgery, The Third Hospital of Hebei Medical University, 050051 Shijiazhuang, Hebei China

**Keywords:** Hepatocellular carcinoma, Oxidative stress, Super enhancer, NRF1, SPIDR

## Abstract

**Background:**

Cellular response to oxidative stress plays significant roles in hepatocellular carcinoma (HCC) development, yet the exact mechanism by which HCC cells respond to oxidative stress remains poorly understood. This study aimed to investigate the role and mechanism of super enhancer (SE)-controlled genes in oxidative stress response of HCC cells.

**Methods:**

The GSE112221 dataset was used to identify SEs by HOMER. Functional enrichment of SE-controlled genes was performed by Metascape. Transcription factors were predicted using HOMER. Prognosis analysis was conducted using the Kaplan-Meier Plotter website. Expression correlation analysis was performed using the Tumor Immune Estimation Resource web server. NRF1 and SPIDR expression in HCC and normal liver tissues was analyzed based on the TCGA-LIHC dataset. ChIP-qPCR was used to detect acetylation of lysine 27 on histone 3 (H3K27ac) levels of SE regions of genes, and the binding of NRF1 to the SE of *SPIDR*. To mimic oxidative stress, HepG2 and Hep3B cells were stimulated with H_2_O_2_. The effects of NRF1 and SPIDR on the oxidative stress response of HCC cells were determined by the functional assays.

**Results:**

A total of 318 HCC-specific SE-controlled genes were identified. The functions of these genes was significant association with oxidative stress response. *SPIDR* and *RHOB* were enriched in the “response to oxidative stress” term and were chosen for validation. SE regions of *SPIDR* and *RHOB* exhibited strong H3K27ac modification, which was significantly inhibited by JQ1. JQ1 treatment suppressed the expression of SPIDR and RHOB, and increased reactive oxygen species (ROS) levels in HCC cells. TEAD2, TEAD3, NRF1, HINFP and TCFL5 were identified as potential transcription factors for HCC-specific SE-controlled genes related to oxidative stress response. The five transcription factors were positively correlated with SPIDR expression, with the highest correlation coefficient for NRF1. NRF1 and SPIDR expression was up-regulated in HCC tissues and cells. NRF1 activated *SPIDR* transcription by binding to its SE. Silencing SPIDR or NRF1 significantly promoted ROS accumulation in HCC cells. Under oxidative stress, silencing SPIDR or NRF1 increased ROS, malondialdehyde (MDA) and γH2AX levels, and decreased superoxide dismutase (SOD) levels and cell proliferation of HCC cells. Furthermore, overexpression of SPIDR partially offset the effects of NRF1 silencing on ROS, MDA, SOD, γH2AX levels and cell proliferation of HCC cells.

**Conclusion:**

NRF1 driven *SPIDR* transcription by occupying its SE, protecting HCC cells from oxidative stress-induced damage. NRF1 and SPIDR are promising biomarkers for targeting oxidative stress in the treatment of HCC.

**Supplementary Information:**

The online version contains supplementary material available at 10.1186/s12876-024-03183-1.

## Introduction

Hepatocellular carcinoma (HCC) is a primary malignant tumor originated from hepatocytes and the most frequent subtype of primary liver cancer [1, 2]. Surgical resection is the mainstay treatment for patients with early-stage HCC [2]. However, most HCC patients are asymptomatic in the early stages of the disease [2]. Treatment strategies for patients with advanced HCC mainly include chemotherapy, radiotherapy, immunotherapy and molecular targeted therapies [3–6]. Despite great efforts in the treatment of HCC, the 5-year survival rate of HCC is still poor due to its high recurrence rate and metastasis [7]. Therefore, it is urgent to discover novel therapeutic targets for HCC.

Oxidative stress refers to the state of cellular stress caused by the imbalance of redox status, which is conducive to promoting oxidation conditions [8]. Factors such as radiation, heat stress and chemotherapy can trigger cellular oxidative stress [9, 10]. Increased reactive oxygen species (ROS) production and decreased antioxidant ability are the major causes of cellular oxidative stress. Excessive accumulation of ROS can cause serious damage to tumor cells, such as DNA double-strand breaks, resulting in tumor cell death [11–15]. Tumors need to overcome high levels of ROS to maintain tumor cell growth and proliferation [14, 15]. However, the mechanism by which HCC cells respond to oxidative stress remains elusive.

The concept of “super enhancers (SEs)” was originally proposed in embryonic stem cells [16]. Recently, mounting evidence has indicated that SEs are associated with widespread activation of multiple oncogenes in tumor cells and facilitate tumor development by contributing to the adaptation of tumor cells to the tumor microenvironment [17, 18]. Different from typical enhancers (TEs), SEs are clusters of multiple adjacent transcriptionally active enhancers that synergistically drive the transcription of target genes [19]. SEs are typically surrounded by nucleosomes with high levels of acetylation of lysine 27 on histone 3 (H3K27ac). Based on this feature, SEs can be predicted by particularly dense and long stretches of H3K27ac signals [16, 19, 20]. SEs need to bind to transcription factors and cofactors, such as bromodomain-containing protein 4 (BRD4), to fulfill their transcriptional activation function [17, 18, 21]. Exploring transcription factors that bind to SEs could help to understand the mechanism by which SEs activate transcription of target genes. HCC cells exhibit a specific SE landscape distinct from normal hepatocytes [21]. As important c*is*-regulatory elements, SEs play crucial roles in regulating the malignant phenotype of HCC such as epithelial-mesenchymal transition, invasion and metastasis [22–24]. However, the mechanism by which SEs regulate the oxidative stress response of HCC are still poorly understood.

In the current study, we aimed to investigate the role and mechanism of SE-controlled genes in oxidative stress response of HCC. To achieve this, we identified SE-controlled genes specific to HCC, and analyzed the oxidative stress response-related genes among them. Subsequently, we screened for key transcription factor that regulates the above genes and significantly affects the survival of HCC patients. Finally, we conducted in vitro experiments to elucidate the regulation of oxidative stress response-related SE-controlled gene by the key transcription factor, and analyzed their effects on oxidative stress response and proliferation of HCC cells.

## Materials and methods

### Identification of SE and SE-controlled genes

H3K27ac chromatin immunoprecipitation-sequencing (ChIP-seq) data from the GSE112221 dataset was employed for SEs identification. Enhancer score was calculated using the findPeaks function available in HOMER [25]. Enhancers were ranked according to enhancer scores using the super style in the findPeaks function in HOMER. Enhancers within a 12.5 kb region were stitched together. SE was defined as the region with a slope greater than 1, while TE was defined as the region with a slope less than or equal to 1. H3K27ac peaks were visualized by the Integrative Genomics Viewer (IGV; https://igv.org). SE-controlled genes were identified as the genes with transcription start sites closest to the center of SEs using the annotatePeaks function in HOMER.

### Functional enrichment analysis

Functional analysis of HCC-specific SE-controlled genes was performed using the Metascape website (http://metascape.org/) with default parameters.

### Transcription factor motif prediction and prognostic analysis

*De novo* motif prediction was performed using HOMER. Recurrence-free survival (RFS) curves were generated using the Kaplan-Meier Plotter online tool (http://kmplot.com/analysis/) with default parameters.

### Cell culture and treatment

Human HCC cells, HepG2 and Hep3B, were purchased from American Type Culture Collection (ATCC; USA). Normal human hepatocytes, L02, were purchased from Cell Bank of the Chinese Academy of Sciences (Shanghai, China). All cells were cultured in Dulbecco’s modified Eagle’s medium (DMEM; Solarbio, China) supplemented with 10% FBS and 1% penicillin-streptomycin at 37˚C with 5% CO_2_. For JQ1 treatment, HepG2 and Hep3B cells were treated with 2, 5 and 10 µM JQ1 (Sigma-Aldrich, USA) or DMSO (Solarbio, China) for 48 h. For H_2_O_2_ stimulation, HepG2 and Hep3B cells were treated with 50 µM H_2_O_2_ for 30 min.

### RNA interference and gene overexpression

Small interfering RNA (siRNA) targeting NRF1 (siNRF1), siRNA targeting SPIDR (siSPIDR), siRNA negative control (siNC), SPIDR overexpression plasmid (pcSPIDR) and the negative control plasmid (pcDNA3.1) were purchased from GenePharma (Shanghai, China). SiNRF1, siSPIDR, siNC, pcSPIDR or pcDNA3.1 was transfected into HepG2 and Hep3B cells using Lipofectamine 3000 (Invitrogen, USA) according to the manufacturer’s instructions.

### Quantitative real-time PCR (qRT-PCR)

Total RNA from L02, HepG2 and Hep3B cells was extracted using TRIzol reagent (Invitrogen, USA), followed by reverse transcription to generate cDNA using the High-Capacity cDNA Reverse Transcription Kit (Applied Biosystems, USA) according to the manufacturer’s instructions. Quantitative PCR was carried out using the SYBR Premix Ex Taq kit (Takara, China) on an ABI 7500 system (Applied Biosystems, USA). Sequences of primers for qRT-PCR were shown in Table 1. GAPDH was selected as the endogenous control. Relative expression levels were calculated according to 2^−∆∆Ct^ method.

**Table 1:** Primer sequences for qRT-PCR.

Gene	Primer sequence (5’-3’)	
RHOB	Forward	ATCCCCGAGAAGTGGGTCC
	Reverse	CGAGGTAGTCGTAGGCTTGGA
SPIDR	Forward	GCTCGGGGCTCTAAGAGAAAA
	Reverse	TGACGGATTCCCAGAAGTGTT
NRF1	Forward	GCTGATGAAGACTCGCCTTCT
	Reverse	TACATGAGGCCGTTTCCGTTT
GAPDH	Forward	GGAGCGAGATCCCTCCAAAAT
	Reverse	GGCTGTTGTCATACTTCTCATGG

### Western blotting

L02, HepG2 and Hep3B cells were collected and lysed with RIPA buffer (Sigma-Aldrich, USA). Lysates were quantified using the Pierce bicinchoninic acid (BCA) Protein Assay Kit (Thermo Fisher Scientific, USA) according to the manufacturer’s instructions. 30 µg extracted protein were loaded into each lane of the SDS-PAGE gel for separation. The separated proteins were transferred onto polyvinylidene difluoride (PVDF) membranes (Millipore, USA), followed by blocking with 5% skim milk for 45 min at room temperature. Then, the membranes were incubated with primary antibodies at 4 °C overnight. Goat anti-rabbit IgG antibody (1:2000 dilution; ab7090, abcam, USA) was used as the secondary antibody for 2 h incubation at room temperature. GAPDH was selected as an internal control. Immunoreactive bands were visualized by ECL reagent (Amersham Biosciences UK Ltd., UK) and quantified by densitometry using ImageJ software (National Institutes of Health, USA). Primary antibodies were anti-RHOB (1:1000 dilution; ab277779, abcam, USA), anti-NRF1 (1:1000 dilution; ab34682, abcam, USA), anti-SPIDR (1:1000 dilution; HPA041582, Sigma-Aldrich, USA) and anti-GAPDH (1:1000 dilution; ab8245, abcam, USA).

### Chromatin immunoprecipitation (ChIP) assay

L02, HepG2 and Hep3B cells were crosslinked with 1% formaldehyde at room temperature for 10 min, followed by neutralization with glycine for 5 min and lysis with SDS lysis buffer (Beyotime, China). The crosslinked DNA was fragmented into 100–1000 bp fragments using Bioruptor UCD-300 (Diagenode, Belgium). Chromatin immunoprecipitation was performed using the Chromatin Immunoprecipitation (ChIP) Assay Kit (Millipore, USA). Sonicated cell lysate was subjected to immunoprecipitation with anti-H3K27ac (ab4729, abcam, USA), anti-NRF1 (ab175932, abcam, USA), anti-histone H3 (ab1791, abcam, USA) and anti-IgG (3900 S, Cell Signaling Technology, Switzerland). Immunoprecipitated DNA was purified using the Gel Extraction Kit (Omega Bio-tek, USA) and subjected to quantification by qRT-PCR. Primer sequences were provided in Table 2.

**Table 2:** Primer sequences for ChIP-qPCR.

Gene	SE region	Primer sequence (5’-3’)	
RHOB	SE1	Forward	GCACAAGACTCCCTCCCTTC
		Reverse	AGGTTCCTCCAACCCTGAGA
	SE2	Forward	CCAGTCTGAGGGAAGCACAG
		Reverse	CCTCAGTTCCACACTTCGCT
	SE3	Forward	ACCAAGGGAGACCAGGTTGT
		Reverse	GTGACCCCACACCAACGATT
SPIDR	SE1	Forward	CACAGACACTGCTTTGGTGC
		Reverse	CACAGCACTGGGACTTTTGC
	SE2	Forward	ACTCCAGAAACTGGGCATCG
		Reverse	AGGCACAGCCCCTAGTGATA
	SE3	Forward	TGCGCATTTGTCTGGTAGGT
		Reverse	TCTTGAGCTAAGGCTTGGGC
	SE4	Forward	GATACACGTAGCAGCCGTGA
		Reverse	TTCTCCCGAAGAACGCAGAC

### ROS assay

Intracellular ROS levels of HepG2 and Hep3B cells were detected using the ROS Assay Kit (Beyotime, China) according to the manufacturer’s instructions by a microplate reader (PerkinElmer, USA) at 485 nm excitation and 535 nm emission wavelength.

### Cell counting Kit-8 (CCK-8) assay

HepG2 and Hep3B cells were seeded into 96-well plates. Cell proliferation was measured on the following 0, 1, 2 and 3 days using the Cell Counting Kit-8 (Beyotime, China). 10 µL of CCK-8 solution was added to each well and incubated at 37 °C for 2 h. The optical density (OD) at 450 nm was measured using a microplate reader (PerkinElmer, USA).

### Enzyme-linked immunosorbent assay (ELISA)

Malondialdehyde (MDA) and superoxide dismutase (SOD) levels were measured using the MDA Assay Kit (Jiancheng Bioengineering, China) and the SOD Assay Kit (Jiancheng Bioengineering, China), respectively.

### Immunofluorescence

HepG2 and Hep3B cells were plated onto glass coverslips coated with gelatin in 24-well plates. Then, cells were fixed with 4% paraformaldehyde for 15 min, permeabilized with 0.25% Triton-X 100 for 15 min, and blocked with 5% BSA in PBS for 30 min. Subsequently, cells were incubated with anti-γH2AX antibody (1:1000 dilution, ab229914, abcam, USA) for 1.5 h, followed by incubation with Alexa Fluor® 647 conjugated goat anti-rabbit IgG (1:1000 dilution, ab150079, abcam, USA) for 1 h. Nuclear was counterstained with 1 µg/mL DAPI solution (Thermo Fisher Scientific, USA). Stained cells were visualized with a Zeiss LSM700 confocal microscope (Carl Zeiss, Germany).

### Statistical analysis

Statistical analysis was performed using GraphPad Prism 9.0 and R version 4.0.2. Comparisons among more than two groups were performed using one-way analysis of variance (ANOVA) with Tukey’s post hoc test. Comparisons between two groups were performed using Student’s t-test. Correlation analysis of gene expression was performed using the Tumor Immune Estimation Resource (TIMER; cistrome.shinyapps.io/timer). *P* < 0.05 was considered to represent a significant difference.

## Results

### Screening of HCC-specific SE-controlled genes

**Fig. 1:**
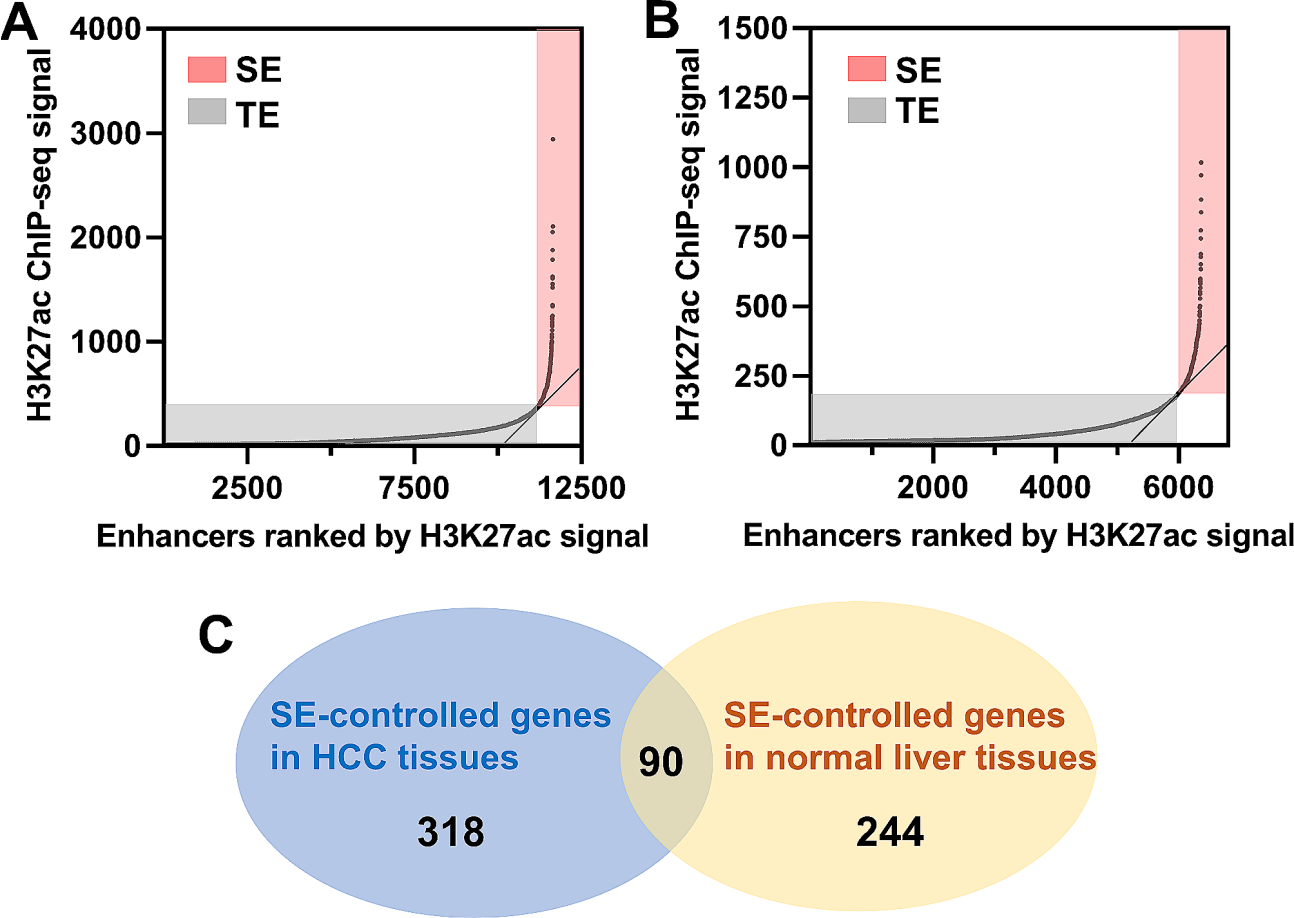
Identification of SE-controlled genes specific in HCC. (**A**-**B**), enhancers in HCC (**A**) and normal liver tissues (**B**) were plotted according to H3K27ac signals based on the GSE112221 dataset. Enhancers with a slope greater than 1 (red) were classified as super enhancers (SEs), while those with a slope less than or equal to 1 (grey) were termed typical enhancers (TEs). (**C**), Venn diagram of SE-controlled genes in HCC and normal liver tissues.

To reveal the role of SEs in HCC progression, we attempted to identify HCC-specific SEs. The GSE112221 dataset, containing H3K27ac ChIP-seq data from HCC and normal liver tissues, was used for enhancer identification. A total of 11,655 enhancers including 433 SEs and 11,222 TEs were identified in HCC tissues (Fig. 1A). As for normal liver tissues, 6,372 enhancers including 346 SEs and 6,026 TEs were recognized (Fig. 1B). To determine genes regulated by SEs, we assigned SEs to the nearest genes and designated these genes as SE-controlled genes. As shown in the Venn diagram, 408 SE-controlled genes were identified in HCC tissues and 334 in normal liver tissues (Fig. 1C). Overlapping analysis showed that 90 SE-controlled genes were shared between normal liver tissues and HCC tissues (Fig. 1C). A total of 318 SE-controlled genes were specific in HCC tissues and 244 SE-controlled genes were specific in normal liver tissues (Fig. 1C). Taken together, 318 HCC-specific SE-controlled genes were filtered.

### HCC-specific SE landscape was associated with oxidative stress response

**Fig. 2:**
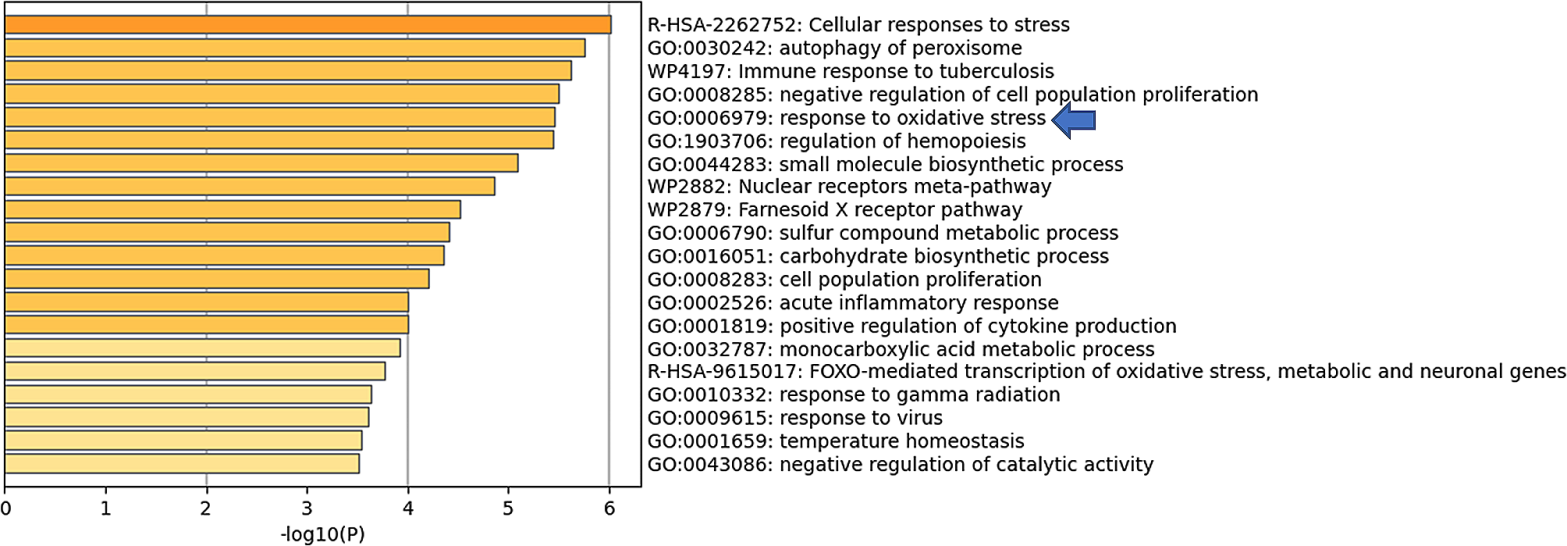
Functional enrichment analysis of HCC-specific SE-controlled genes by the Metascape online tool.

Functional enrichment analysis of the 318 HCC-specific SE-controlled genes was performed using the Metascape online tool. The enriched pathways mainly included processes associated with cellular stress response (e.g. response to oxidative stress, and response to gamma radiation), cellular metabolism (e.g. sulfur compound metabolic process, monocarboxylic acid metabolic process, and carbohydrate biosynthetic process) and cell proliferation (Fig. 2). Overcoming oxidative stress is essential for the survival of tumor cells [26, 27]. In this study, we focused on the eight genes enriched in the “response to oxidative stress” category (*GPR98*, *SPIDR*, *TAT*, *DHCR24*, *RHOB*, *ZFP36L1*, *MTHFS* and *MBP*).

**Fig. 3:**
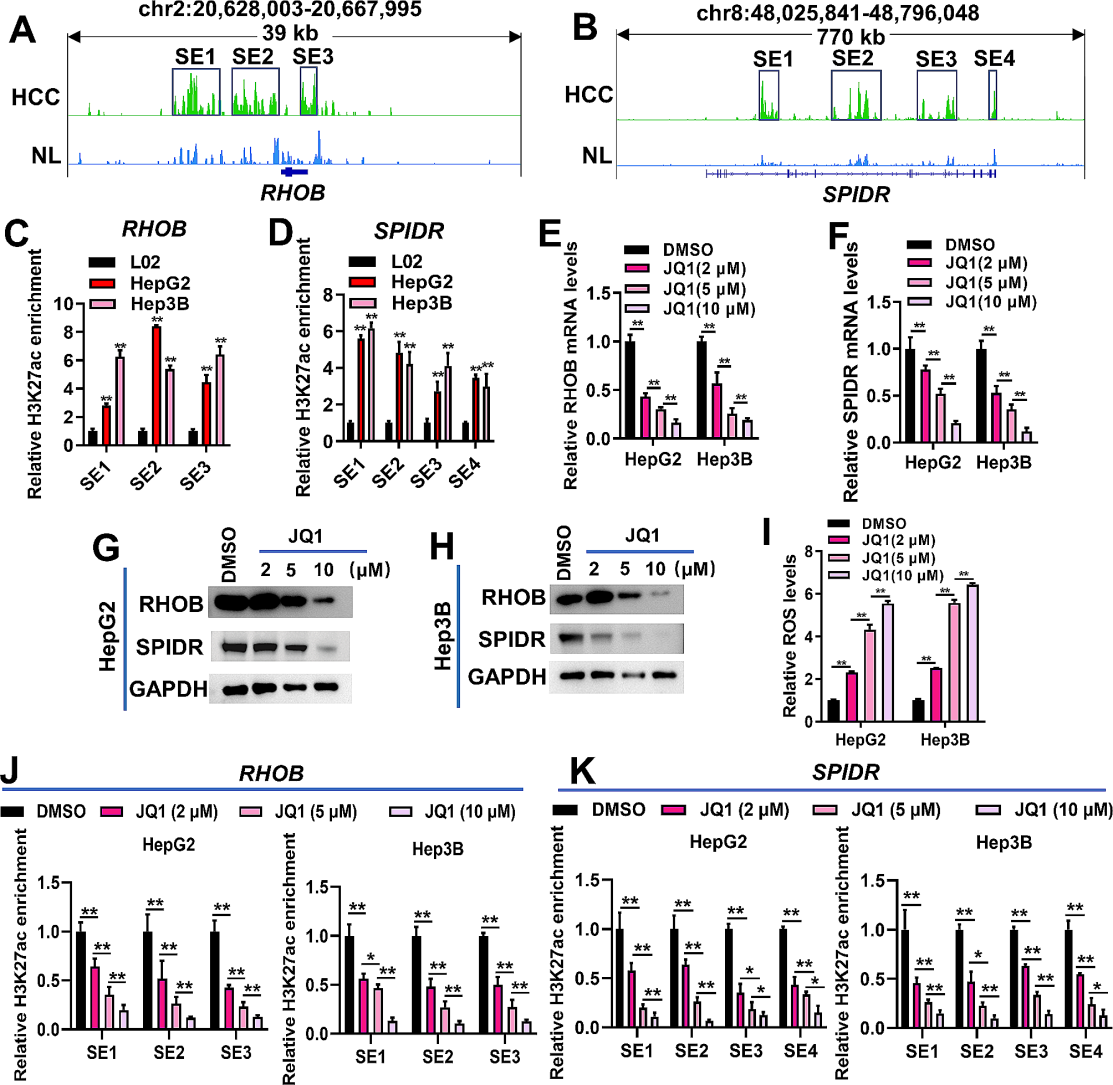
*RHOB* and *SPIDR* were selected from genes enriched in the “response to oxidative stress” term for verification. (**A**-**B**), analysis of H3K27ac signals at the *RHOB* (**A**) and *SPIDR* (**B**) loci in HCC and normal liver (NL) tissues based on the GSE112221 dataset. The SE of *RHOB* was divided into three constituents (SE1, SE2 and SE3). The SE of *SPIDR* was divided into four constituents (SE1, SE2, SE3 and SE4). (**C**-**D**), ChIP-qPCR was used to detect the H3K27ac levels at the SE constituents of *RHOB* (**C**) and *SPIDR* (**D**) loci in L02, HepG2 and Hep3B cells. **P < 0.01, vs. L02. (**E**-**H**), qRT-PCR and Western blotting were employed to detect the mRNA (**E**-**F**) and protein (**G**-**H**) expression of RHOB and SPIDR in HepG2 and Hep3B cells, respectively. HepG2 and Hep3B cells were treated with DMSO or JQ1 (2, 5 and 10 µM). **P < 0.01. (**I**), effect of JQ1 on ROS levels of HepG2 and Hep3B cells. **P < 0.01. (**J**-**K**), ChIP-qPCR was used to detect the H3K27ac levels at the SE constituents of *RHOB* (**J**) and *SPIDR* (**K**) in HepG2 and Hep3B cells treated with DMSO or JQ1 (2, 5 and 10 µM). *P < 0.05, **P < 0.01. Data for C-F and I-K were shown as mean ± SD of three independent experiments.

In order to further demonstrate that genes enriched in the “response to oxidative stress” category are regulated by SEs and affect cellular oxidative stress, we selected *RHOB* and *SPIDR*, which are involved in cell stress response, for validation [28, 29]. Based on the ChIP-seq data from GSE112221, we found that H3K27ac signals around the *RHOB* and *SPIDR* loci were obviously stronger in HCC than in normal liver tissues (Fig. 3A-B). The SE of *RHOB* was divided into three constituents (SE1, SE2 and SE3), and the SE of *SPIDR* was divided into four constituents (SE1, SE2, SE3 and SE4) (Fig. 3A-B). ChIP-qPCR results demonstrated that H3K27ac levels of the three SE constituents of *RHOB* and the four SE constituents of *SPIDR* were significantly up-regulated in HepG2 and Hep3B cells compared to L02 cells, which confirmed the ChIP-seq results (Fig. 3C-D). We stimulated HCC cells with gradient concentrations of JQ1 (a BRD4 inhibitor) to disrupt SE activity [30]. JQ1 treatment inhibited the mRNA and protein expression of RHOB and SPIDR in HepG2 and Hep3B cells (Fig. 3E-H). JQ1 stimulation up-regulated the accumulation of ROS in HCC cells, and the up-regulation was enhanced with increasing JQ1 concentration (Fig. 3I). Additionally, JQ1 treatment significantly down-regulated H3K27ac enrichment in the SE constituents of *RHOB* and *SPIDR* in a dose-dependent manner (Fig. 3J-K). Overall, these results suggested that genes controlled by HCC-specific SEs were associated with oxidative stress response of HCC cells.

### NRF1 was a potential transcription factor for *SPIDR*

**Fig. 4:**
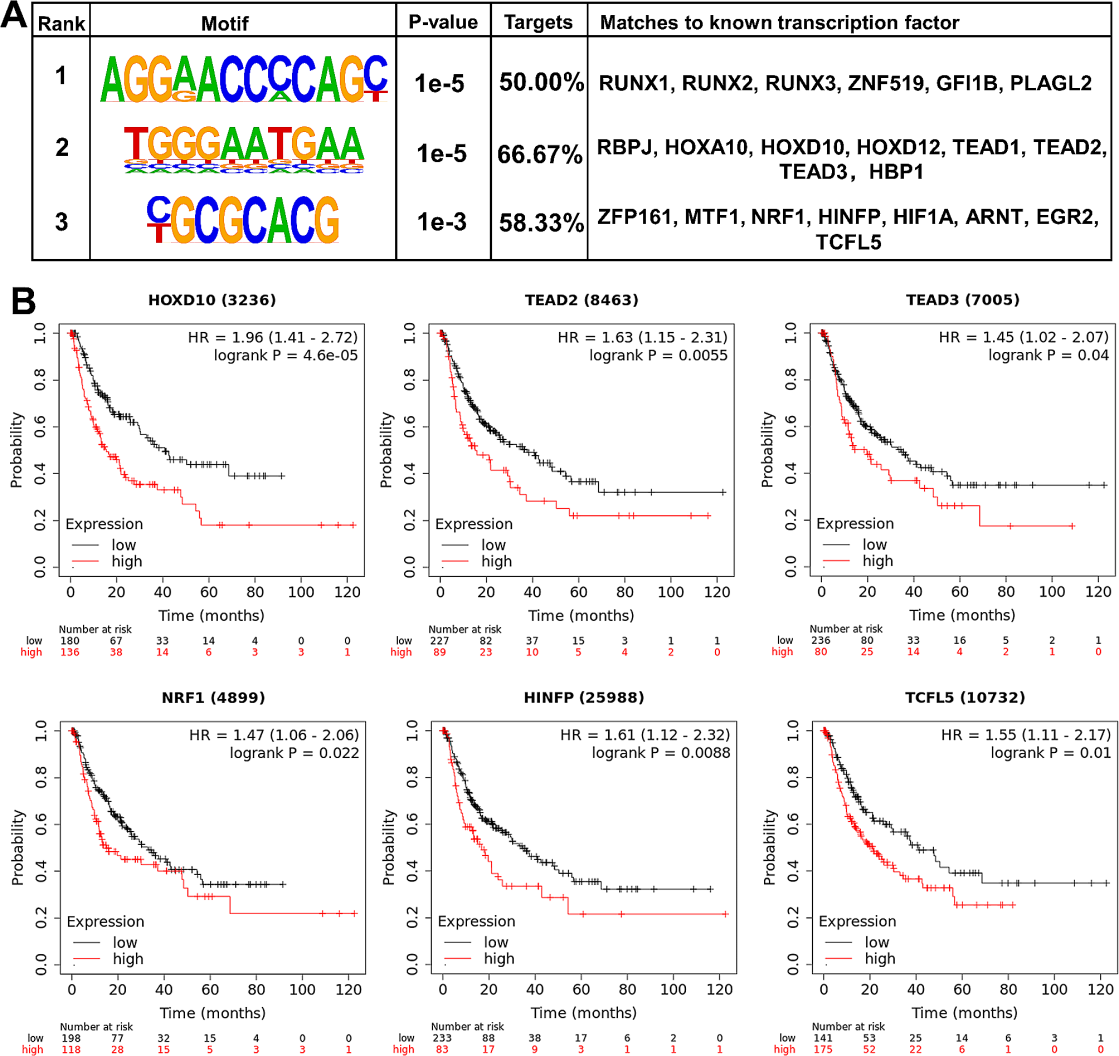
Prediction and prognostic analysis of transcription factors for HCC-specific SE-controlled genes related to oxidative stress response. (**A**), schematic images of the top three representative enriched transcription factor-binding motifs and the corresponding matched transcription factors. (**B**), high expression of HOXD10, TEAD2, TEAD3, NRF1, HINFP and TCFL5 corresponds to a poor recurrence-free survival of HCC patients. Prognostic analysis was performed using the Kaplan-Meier Plotter website.

To investigate the regulatory mechanism underlying HCC-specific SE-controlled genes related to oxidative stress response, we employed HOMER to analyze the transcription factor-binding motifs of the eight such genes (*GPR98*, *SPIDR*, *TAT*, *DHCR24*, *RHOB*, *ZFP36L1*, *MTHFS* and *MBP*). The top three motifs matched 22 transcription factors (Fig. 4A). It was found that the top motif corresponded to six transcription factors, including RUNX1, RUNX2, RUNX3, ZNF519, GFI1B and PLAGL2 (Fig. 4A). The second motif matched eight transcription factors, including RBPJ, HOXA10, HOXD10, HOXD12, TEAD1, TEAD2, TEAD3 and HBP1 (Fig. 4A). The third motif matched eight transcription factors, including ZFP161, MTF1, NRF1, HINFP, HIF1A, ARNT, EGR2 and TCFL5 (Fig. 4A).

Subsequently, we analyzed the prognostic value of the 22 potential transcription factors using the Kaplan-Meier Plotter website. A poor RFS was observed in HCC patients with high expression of HOXD10, TEAD2, TEAD3, NRF1, HINFP and TCFL5 (Fig. 4B). Conversely, low expression of RUNX2, RUNX3, GFI1B, EGR2, HOXD12 and HIF1A was significantly correlated with a poor RFS of HCC patients (Figure S1A). The expression of RUNX1, ZNF519, PLAGL2, RBPJ, HOXA10, TEAD1, ZFP161, ARNT, MTF1 and HBP1 had no significant impact on RFS of HCC patients (Figure S1B).

We evaluated the correlation among the expression of the six poor prognostic transcription factors (HOXD10, TEAD2, TEAD3, NRF1, HINFP and TCFL5) and the eight oxidative stress response-related SE-controlled genes (*GPR98*, *SPIDR*, *TAT*, *DHCR24*, *RHOB*, *ZFP36L1*, *MTHFS* and *MBP*) using TIMER (cistrome.shinyapps.io/timer). Cor > 0.2 and *P* < 0.05 was considered to be significant positive correlation. Except for HOXD10, the expression of the remaining five transcription factors (TEAD2, TEAD3, NRF1, HINFP and TCFL5) were positively correlated with at least one oxidative stress response-related SE-controlled genes (Table 3). Of particular note, all of the five transcription factors were positively correlated with SPIDR expression, with correlation coefficients of NRF1 (cor = 0.519), HINFP (cor = 0.467), TEAD3 (cor = 0.448), TEAD2 (cor = 0.323) and TCFL5 (cor = 0.291) in descending order (Table 3).

**Table 3:** Transcription factors that were positively correlated with the oxidative stress response-related SE-controlled genes

Transcription factor	Oxidative stress response-related SE-controlled gene	Cor	*P*-value
TEAD2	SPIDR	0.323	1.85e-10
TEAD3	SPIDR	0.448	0.00
	ZFP36L1	0.328	1.29e-10
	MBP	0.386	1.37e-14
NRF1	SPIDR	0.519	0.00
	ZFP36L1	0.385	1.58e-14
	MBP	0.42	0.00
HINFP	SPIDR	0.467	0.00
	DHCR24	0.295	8.64e-09
	ZFP36L1	0.371	1.91e-13
	MBP	0.438	0.00
TCFL5	SPIDR	0.291	1.39e-08
	DHCR24	0.365	5.51e-13
	ZFP36L1	0.294	9.71e-09
	MBP	0.349	6.47e-12

Taken together, TEAD2, TEAD3, NRF1, HINFP and TCFL5 were identified as potential transcription factors for the oxidative stress response-related SE-controlled genes, which expression were detrimental to RFS of HCC patients. *SPIDR* was a potential target gene common to the above five transcription factors, and was most strongly correlated with the expression of NRF1. Therefore, we selected NRF1 and SPIDR for the follow-up study.

### The expression of NRF1 and SPIDR were up-regulated in HCC tissues and cells

**Fig. 5:**
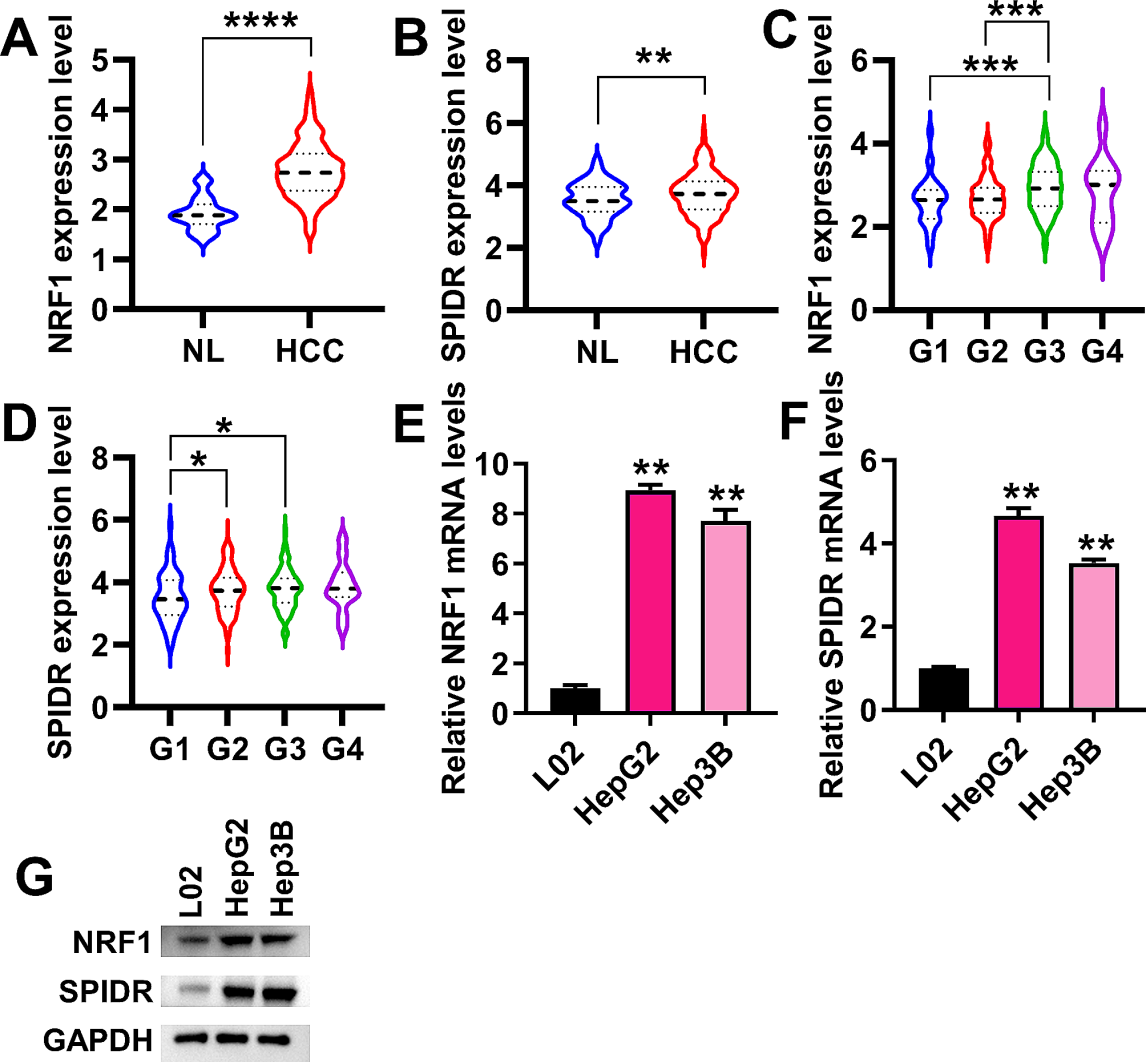
Clinical feature analysis of NRF1 and SPIDR expression. (**A**-**B**), NRF1 (**A**) and SPIDR (**B**) expression in HCC and normal liver (NL) tissues was analyzed based on the TCGA-LIHC dataset. NL, *n* = 50. HCC, *n* = 371. **P < 0.01, ****P < 0.0001. (**C**-**D**), NRF1 (**C**) and SPIDR (**D**) expression in different HCC grades was analyzed based on the TCGA-LIHC dataset. G1, *n* = 55. G2, *n* = 177. G3, *n* = 122. G4, *n* = 12. *P < 0.05, ***P < 0.001. (**E**-**F**), qRT-PCR was performed to detect the relative mRNA expression of NRF1 (**E**) and SPIDR (**F**) in L02, HepG2 and Hep3B cells. **P < 0.01. (**G**), Western blotting was used to detect the protein levels of NRF1 and SPIDR in L02, HepG2 and Hep3B cells. Data for A-D were shown as median ± SD. Data for E-F were shown as mean ± SD of three independent experiments.

The expression of NRF1 and SPIDR in HCC and normal liver tissues were analyzed based on The Cancer Genome Atlas (TCGA)-LIHC dataset. It was found that NRF1 and SPIDR were up-regulated in HCC tissues compared with normal liver tissues (Fig. 5A-B). Then, we analyzed the expression of NRF1 and SPIDR in different tumor grades based on the TCGA-LIHC dataset. The expression of NRF1 showed significant differences in G1 vs. G3, and G2 vs. G3 (Fig. 5C). There were significant differences in SPIDR expression in G1 vs. G2, and G1 vs. G3 (Fig. 5D). Additionally, qRT-PCR and Western blotting were used to measure NRF1 and SPIDR mRNA and protein expression in normal hepatocytes and HCC cells, respectively. Consistent with the results based on the TCGA-LIHC dataset, NRF1 and SPIDR mRNA and protein levels were up-regulated in HCC cells compared with L02 cells (Fig. 5E-G). Collectively, we found that NRF1 and SPIDR expression were up-regulated in HCC tissues and cells.

### SE-controlled *SPIDR* was transcriptionally activated by NRF1

**Fig. 6:**
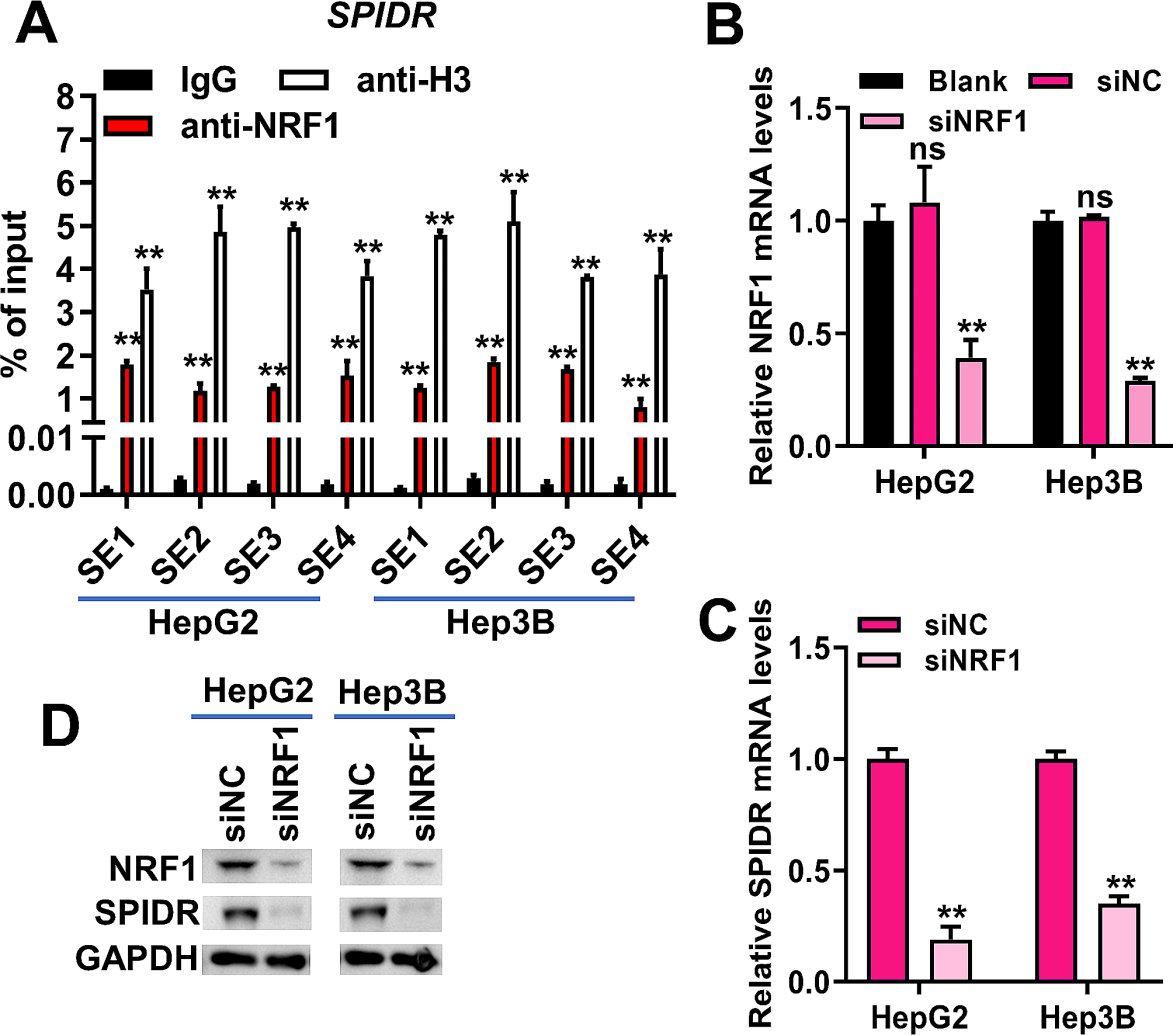
NRF1 bound to the SE of *SPIDR* to drive its transcription. (**A**), ChIP-qPCR was used to measure the binding between NRF1 and the four SE constituents of *SPIDR*. **P < 0.01 vs. IgG. (**B**-**C**), qRT-PCR was used to detect the mRNA levels of NRF1 (**B**) and SPIDR (**C**) in HepG2 and Hep3B cells transfected with siNC or siNRF1. **P < 0.01 vs. siNC group. Ns, non-significant vs. blank group. (**D**), Western blotting was used to detect the protein levels of NRF1 and SPIDR in HepG2 and Hep3B cells transfected with siNC or siNRF1. Data for A-C were shown as mean ± SD of three independent experiments.

Considering NRF1 was a potential transcription factor regulating the expression of SPIDR, we sought to validate the regulatory role of NRF1 on SPIDR expression. ChIP-qPCR results indicated that anti-NRF1 was significantly enriched in the four SE constituents of *SPIDR* in HepG2 and Hep3B cells, suggesting that NRF1 bound to the SE of *SPIDR* (Fig. 6A). Additionally, NRF1 was successful silenced in HepG2 and Hep3B cells by transfection with siNRF1 (Fig. 6B and D). As expected, silencing of NRF1 decreased SPIDR mRNA and protein levels in HepG2 and Hep3B cells (Fig. 6C-D). Summing up, NRF1 activated *SPIDR* transcription by binding to its SE.

### NRF1 regulated SPIDR to protect HCC cells from oxidative stress-induced damage

**Fig. 7:**
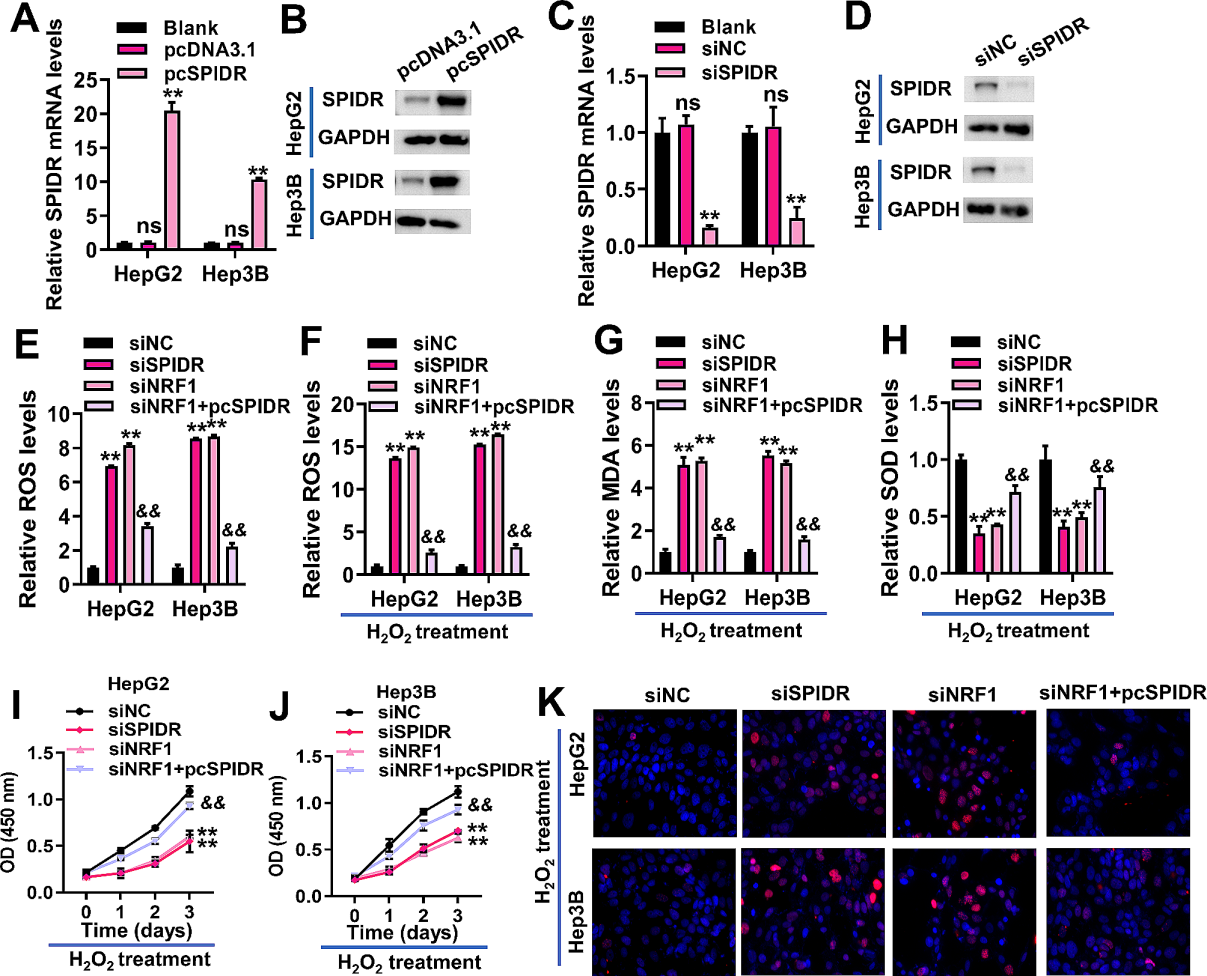
NRF1 regulated SPIDR to protect HCC cells from oxidative stress-induced damage. (**A**-**B**), qRT-PCR and Western blotting were employed to measure SPIDR mRNA (**A**) and protein (**B**) expression in HepG2 and Hep3B cells transfected with pcDNA3.1 or pcSPIDR. **P < 0.01 vs. pcDNA3.1 group. Ns, non-significant vs. blank group. (**C**-**D**), qRT-PCR and Western blotting were employed to measure SPIDR mRNA (**C**) and protein (**D**) expression in HepG2 and Hep3B cells transfected with siNC or siSPIDR. **P < 0.01 vs. siNC group. Ns, non-significant vs. blank group. HepG2 and Hep3B cells transfected with siNC, siSPIDR, siNRF1 or co-transfected with siNRF1 and pcSPIDR were used to detect ROS, MDA, SOD and γH2AX levels, as well as cell proliferation. (**E**-**F**), quantification of ROS in HepG2 and Hep3B cells without (**E**) or with (**F**) H_2_O_2_ treatment. (**G**-**H**), quantification of MDA (**G**) and SOD (**H**) in HepG2 and Hep3B cells with H_2_O_2_ treatment. (**I**-**J**), the proliferation of H_2_O_2_-stimulated HepG2 (**I**) and Hep3B (**J**) cells was detected by CCK-8. **P < 0.01 vs. siNC group. &&*P* < 0.01 vs. siNRF1 group. (**K**), representative immunofluorescence images of γH2AX foci in HepG2 and Hep3B cells stimulated with H_2_O_2_. Red, γH2AX. Blue, nuclear. Data for A, C and E-J were shown as mean ± SD of three independent experiments.

We further investigated the roles of NRF1 and SPIDR in the oxidative stress response of HCC cells. The overexpression and silencing efficiency of SPIDR in HepG2 and Hep3B cells were verified by qRT-PCR and Western blotting (Fig. 7A-D). The effects of NRF1 and SPIDR expression on ROS levels in HCC cells were analyzed. ROS levels were significantly increased with SPIDR or NRF1 silencing in HepG2 and Hep3B cells (Fig. 7E). However, transfection with pcSPIDR partially offset the increased ROS levels induced by NRF1 silencing in HepG2 and Hep3B cells (Fig. 7E).

Then, we mimicked oxidative stress in HepG2 and Hep3B cells by treating cells with H_2_O_2_. Under oxidative stress condition, silencing of NRF1 or SPIDR significantly increased ROS and MDA levels, and significantly decreased SOD levels in HepG2 and Hep3B cells (Fig. 7F-H). Overexpression of SPIDR attenuated the elevation of ROS and MDA levels induced by NRF1 silencing, as well as the reduction of SOD levels in HepG2 and Hep3B cells (Fig. 7F-H). CCK-8 assays showed that under oxidative stress, silencing of NRF1 or SPIDR significantly inhibited the proliferation of HepG2 and Hep3B cells (Fig. 7I-J). However, the inhibition of cell proliferation caused by NRF1 silencing was partially counteracted by SPIDR overexpression (Fig. 7I-J). Oxidative stress leads to DNA double-strand breaks, which is the most harmful type of DNA damage that directly threatens cell survival [31, 32]. In this study, we measured the levels of γH2AX, a marker of DNA double-strand breaks [33]. Immunofluorescence results showed that silencing of SPIDR or NRF1 led to the elevated levels of γH2AX under oxidative stress (Fig. 7K). However, transfection of pcSPIDR reversed the up-regulation of γH2AX caused by NRF1 silencing in HepG2 and Hep3B cells (Fig. 7K). Collectively, these results corroborated that NRF1 regulated SPIDR to protect HCC cells from oxidative stress-induced damage.

## Discussion

In recent years, efforts have been made to elucidate the epigenetic mechanisms that control HCC development, but the prognosis of HCC patients remains poor [7]. Oxidative stress plays an important role in HCC development [34, 35]. In this study, we found that HCC-specific SE-controlled genes were associated with oxidative stress response. Transcription factors that regulate HCC-specific SE-controlled genes were identified. By analyzing the prognostic significance of transcription factors and their correlation with the expression of HCC-specific SE-controlled genes associated with oxidative stress response, we found that NRF1 occupied the SE of *SPIDR*, driving its expression to protect HCC cells from oxidative stress-induced damage.

The continuous and robust transcription of oncogenes driven by SEs promotes the adaptation of tumor cells to the tumor microenvironment, thus promoting tumor development [36, 37]. HCC cells are highly susceptible to the perturbations of SE landscapes [21]. Previous studies have revealed the functions and regulatory mechanisms of many SE-controlled genes in HCC [22, 24, 38, 39]. For example, TCF4 binds to the SE of *AJUBA* and activates its transcription, thereby activating the Akt/GSK-3β/Snail pathway to induce epithelial-mesenchymal transition and invasion of HCC [22]. LncRNA HCCL5, as a SE-controlled oncogenic factor, is regulated by the transcription factor ZEB1 in HCC [24]. HNF4G activates the transcription of SE-controlled lncRNA-DAW and promotes tumor growth by activating Wnt/β-catenin pathway in HCC [38]. However, the functions and regulatory mechanisms of a large number of SE-controlled genes in HCC are still unclear. High levels of H3K27ac modification in the chromatin are a well-recognized signature for SEs identification [16, 19, 20]. In this study, we identified 318 HCC-specific SE-controlled genes. These genes were enriched in biological processes related to cellular stress response, metabolism and cell proliferation, especially in the “response to oxidative stress” term.

Oxidative stress induced by highly toxic ROS is an important contributor hindering tumor development [40, 41]. When ROS accumulated in excess, oxidative stress inhibits tumor cell proliferation and leads to tumor cell senescence and death [40, 41]. Therefore, the mechanisms regulating oxidative stress response of tumor cells have received tremendous attention. For example, the high expression of TRIM25 facilitates the survival of HCC cells by ameliorating oxidative stress through regulating the Keap1-Nrf2 pathway [34]. Overexpression of PCK1 induces oxidative stress and leads to apoptosis of HCC cells under the condition of glucose deprivation [35]. However, the mechanisms regulating oxidative stress response of HCC cells need to be further investigated. In this study, we found that *GPR98*, *SPIDR*, *TAT*, *DHCR24*, *RHOB*, *ZFP36L1*, *MTHFS* and *MBP* were HCC-specific SE-controlled genes significantly enriched in the “response to oxidative stress” term. *RHOB* and *SPIDR* were selected from the above eight genes for verification. Ras homolog family member B (RHOB) is involved in cellular responses to radiation, cisplatin and hydrogen peroxide, all of which could cause cellular oxidative stress [9, 10, 28]. Scaffold protein involved in DNA repair (SPIDR) positively regulate DNA double-strand breaks repair [29]. DNA double-strand breaks can be caused by oxidative stress which is a serious cellular damage [11–13]. We found that the SE regions of *RHOB* and *SPIDR* exhibited strongly enhancement of H3K27ac signals in HCC tissues and cells. To further investigate the role of SEs in regulating the expression of these genes, HCC cells were treated with JQ1. JQ1 is a BRD4 inhibitor which binds to the BET-bromodomain of BRD4, blocking or dissociating the binding of BRD4 to SE regions, thus disrupting the function of SEs and resulting in transcriptional elongation defects of target genes [17]. Our findings demonstrated that JQ1 treatment inhibited the expression of RHOB and SPIDR, as well as down-regulated H3K27ac modification in SEs of these genes in a dose-dependent manner, providing further evidence that *RHOB* and *SPIDR* were regulated by SEs. ROS accumulation is a major driver of oxidative stress [42]. We found that JQ1 treatment significantly increased the ROS levels in HCC cells in a dose-dependent manner.

The intensive occupation of SEs by transcription regulators, such as transcription factors and coactivators, is essential for their transcriptional activation function [17, 18, 21]. In the present study, we found that TEAD2, TEAD3, NRF1, HINFP and TCFL5 were positively correlated with the expression of HCC-specific SE-controlled genes related to oxidative stress response, and the high expression of these transcription factors corresponded to a poor prognosis of HCC patients. Notably, the expression of SPIDR was positively correlated with all of the five transcription factors and showed the strongest correlation with NRF1.

Nuclear respiratory factor 1 (NRF1) is a key factor in the activation of genes involved in the regulation of cellular metabolism and mitochondrial biogenesis [43, 44]. NRF1 promotes cell growth by transcriptionally activating E2F1 in HCC [45]. High expression of NRF1 in HCC tissues is associated with poor prognosis of HCC patients [46]. Consistent with the previous reports [45, 46], our results demonstrated that NRF1 expression was up-regulated in HCC tissues and cells, and its high expression predicted a poor prognosis of HCC patients. SPIDR, also known as KIAA0146, functions in homologous recombination repair of DNA double-strand breaks [31, 47, 48]. However, the role of SPIDR in HCC development has not been well characterized. We found that the expression of SPIDR was significantly up-regulated in HCC tissues and cells. Furthermore, we demonstrated that NRF1 bound to the SE of *SPIDR* to drive its transcription.

To date, the precise functions and regulatory mechanisms of NRF1 and SPIDR in oxidative stress response in HCC are largely unknown. Here, we found that silencing of SPIDR or NRF1 promoted the accumulation of ROS in HCC cells, which can be partially abolished by overexpression of SPIDR. ROS, mainly including superoxide anion (O_2_^•−^), hydrogen peroxide (H_2_O_2_), singlet oxygen (^1^O_2_) and hydroxyl radical (OH^•^), exert harmful effects on cells by damaging macromolecules such as DNA, RNA, lipids and proteins [8, 49]. Among them, H_2_O_2_ is relatively stable and can diffuse freely, with disastrous consequences for cell survival [8, 49]. We mimicked oxidative stress in vitro by treating HCC cells with H_2_O_2_. MDA is an important marker for lipid peroxidation [50, 51]. SOD is an antioxidant enzyme capable of scavenging superoxide anion radicals generated within cells, thereby protecting cells from oxidative damage [52, 53]. Changes in MDA and SOD levels can indicate mitochondrial status [54–57]. Our results showed that under oxidative stress, silencing of SPIDR or NRF1 increased ROS and MDA levels, as well as decreased SOD levels in HCC cells, suggesting that silencing SPIDR or NRF1 impaired the antioxidant capacity of mitochondria. Furthermore, we found that overexpression of SPIDR partially offset the damage to mitochondrial antioxidant capacity caused by NRF1 silencing under oxidative stress. Additionally, silencing of SPIDR or NRF1 hindered the proliferation of HCC cells, which could be partially reversed by overexpression of SPIDR. ROS induces various types of DNA damage, including base oxidation, DNA single-strand breaks and double-strand breaks, with DNA double-strand breaks being particularly detrimental [31, 32]. When DNA undergoes double-strand breaks, the H2AX histones were phosphorylated at the breakage site to generate γH2AX, which serves as a signature of DNA double-strand breaks [33]. Our study revealed that silencing SPIDR or NRF1 increased the levels of γH2AX in HCC cells under oxidative stress condition, which could be partially reversed by overexpression of SPIDR. The complex regulatory mechanisms of NRF1 and SPIDR in the oxidative stress response in HCC, such as the downstream effect factors of the NRF1/SPIDR axis, remain to be further investigated.

## Conclusion

NRF1 was identified as a transcription factor that regulated *SPIDR* expression by binding to its SE, thereby safeguarding HCC cells against oxidative stress. These findings provide valuable insights into the regulation of oxidative stress response in HCC, and hold promise for the development of innovative therapies for HCC.

### Electronic supplementary material

Below is the link to the electronic supplementary material.

Supplementary Material 1

## Data Availability

The datasets used and/or analysed during the current study available from the corresponding author on reasonable request.
