# Supplementary material for: Nuclear respiratory factor 1 regulates super enhancer-controlled SPIDR to protect hepatocellular carcinoma cells from oxidative stress
Source: BMC Gastroenterol. 2024 Mar 4;24:97. doi: 10.1186/s12876-024-03183-1 (PMC10913589; doi:10.1186/s12876-024-03183-1)
Supplement: Supplementary file 1 — Supplementary Material 1 [file 12876_2024_3183_MOESM1_ESM.pdf]

Supplementary information

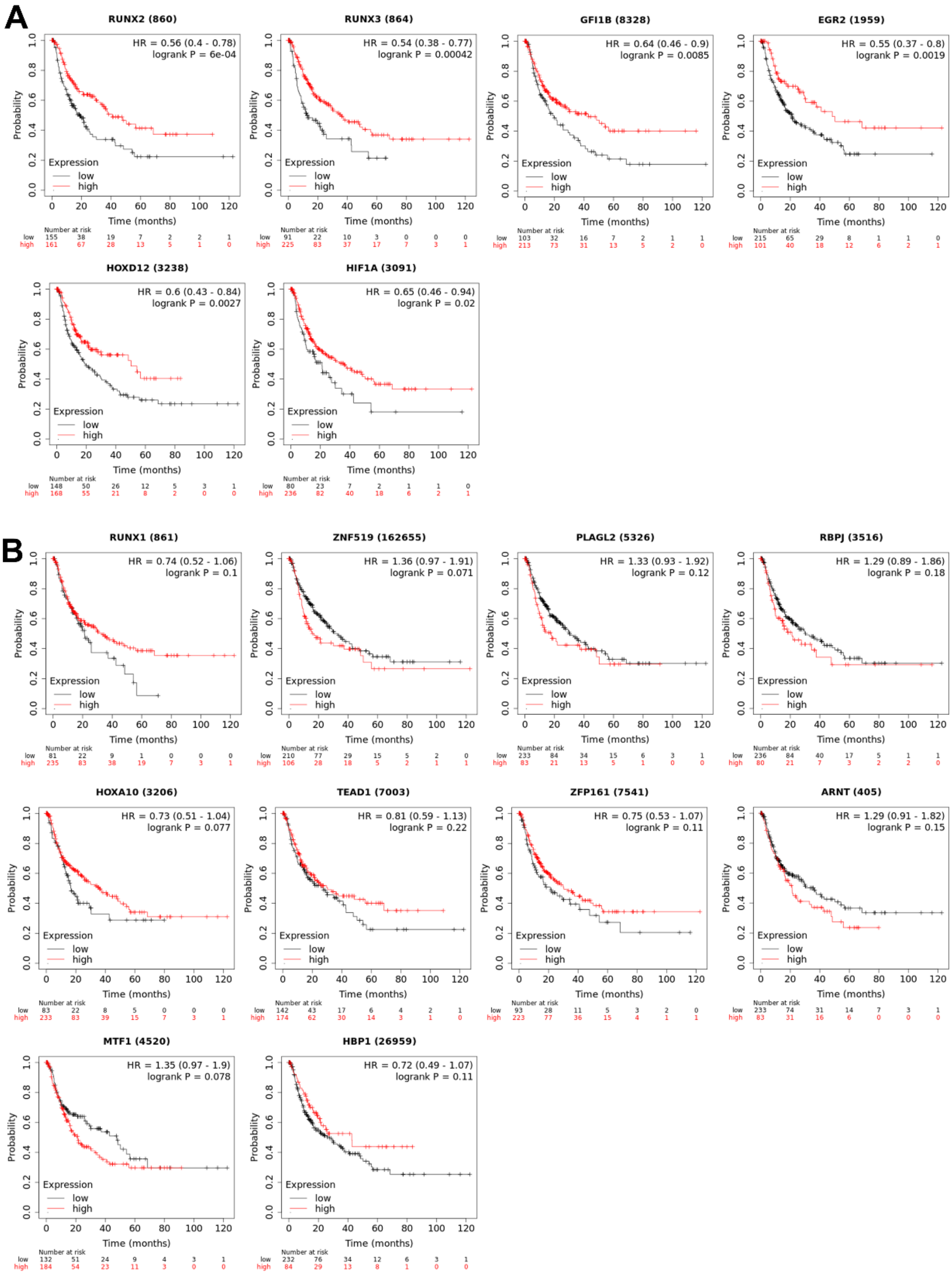

Figure S1 Kaplan-Meier curves for transcription factors. A, transcription factors with low expression corresponding to a poor recurrence-free survival of HCC patients. B, transcription factors with non-significant effect on recurrence-free survival of HCC patients.

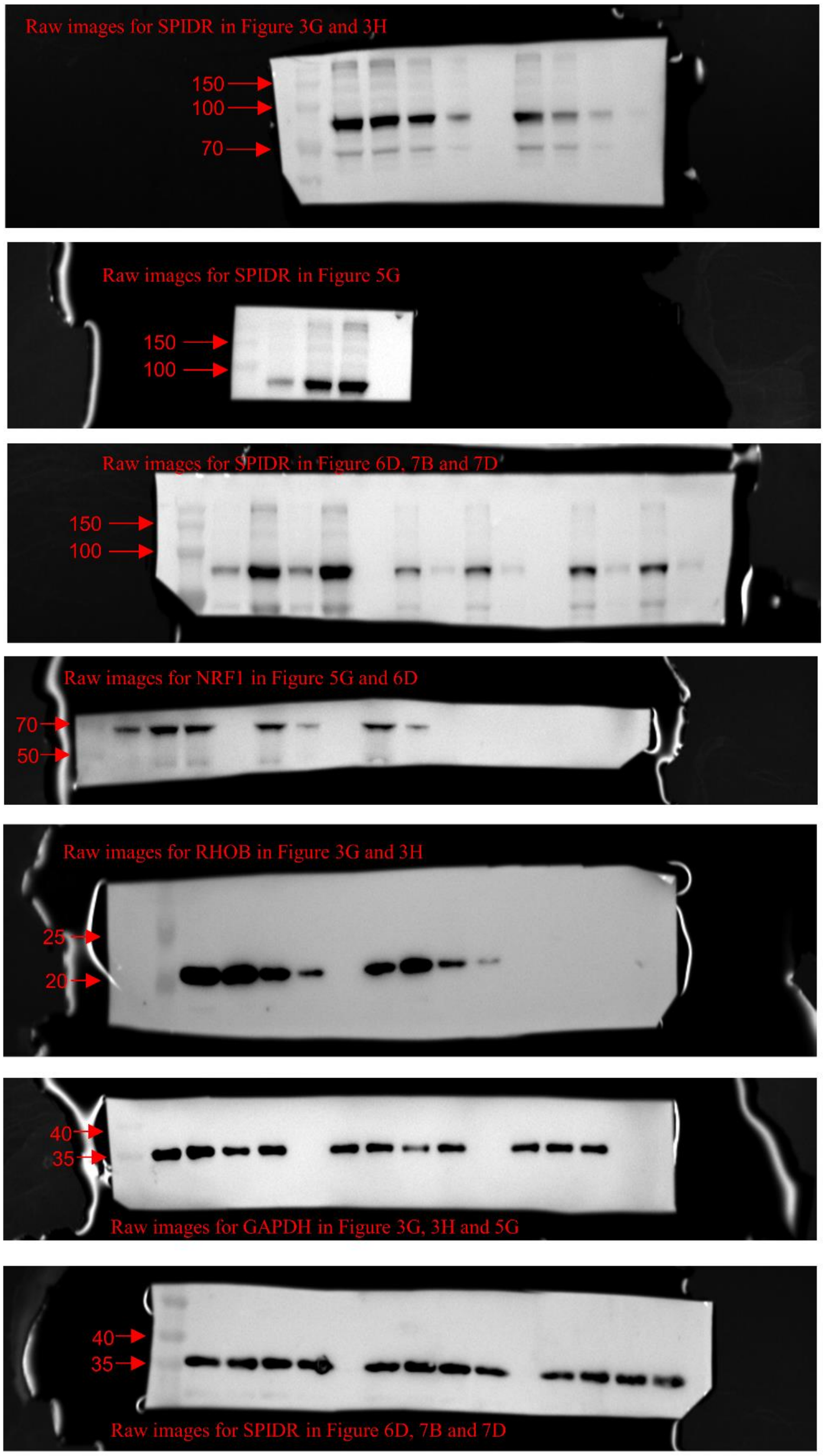

Figure S2 Western blotting raw images. In order to economize the antibodies, we sheared the PVDF membranes according to the number of samples and the molecular weight of the target proteins before the antibodies incubation.
